# Correlation between Colour Traits and Intrinsic Quality of Dalbergiae Odoriferae Lignum

**DOI:** 10.3390/molecules28227635

**Published:** 2023-11-16

**Authors:** Wenjie He, Ying Sun, Sai Zhang, Jiawen Li, Jixing Feng, Yun Yang, Hui Meng, Zheng Zhang

**Affiliations:** 1 Institute of Medicinal Plant Development, Chinese Academy of Medical Sciences and Peking Union Medical College, Beijing 100193, China; z13663786725@163.com (W.H.);; 2 Key Laboratory of Resources Conservation and Development of Southern Medicine of Hainan Province & Hainan Branch of the Institute of Medicinal Plant Development, Chinese Academy of Medical Sciences and Peking Union Medical College, Haikou 570311, China; 3 Hainan Hospital of Chinese PLA General Hospital, Sanya 572000, China

**Keywords:** Dalbergiae Odoriferae Lignum, colour, extract, volatile oil, flavone, quality grade

## Abstract

*Dalbergia odorifera* T. Chen is traditionally referred to as „Dalbergiae Odoriferae Lignum” in traditional Chinese medicine. Its quality is typically assessed subjectively based on colour and texture observations and lacks a universal grading system. Our objective was to establish a relationship between heartwood colour and the content of key constituents, including total flavonoids, six specific flavonoids, alcohol-soluble extracts, and volatile oils, to assess their impact on heartwood quality. Substantial correlations were observed between the colour depth (L*), red–green direction (a*), and yellow–blue direction (b*), as well as the content of the extract, volatile oil, total flavonoids, naringenin, formononetin, pinocembrin, and isoliquiritigenin. Specifically, a* was correlated with the extract, total flavonoids, and isoliquiritigenin, whereas b* was correlated with the extract, volatile oil, total flavonoids, naringenin, formononetin, pinocembrin, and isoliquiritigenin. The results suggested that L*, b*, and chemical composition indices, such as extract, volatile oil, total flavonoids, and naringenin, could serve as primary criteria for classifying the quality of medicinal materials. This is consistent with market classification based on colour and texture, which facilitates material identification and guides the cultivation, harvesting, and processing of *D. odorifera.* This study provides a scientific foundation for its future development and use.

## 1. Introduction

Dalbergiae Odoriferae Lignum, derived from dried heartwood from *Dalbergia odorifera* T. Chen, a leguminous plant renowned for its effects on the resolution of blood stasis, halting bleeding, regulating qi, and alleviating pain, is extensively used in the treatment of conditions such as bruises and angina pectoris due to coronary heart disease [1]. Traditional Chinese medicine is rich in flavonoids and volatile oil, both of which exhibit robust pharmacological activities [2]. Flavonoids within Dalbergiae Odoriferae Lignum possess anti-inflammatory, antioxidant, and analgesic properties, whereas the volatile oil demonstrates antibacterial and antithrombotic activities. These compounds are the main active ingredients of Dalbergiae Odoriferae Lignum [3,4,5,6]. However, quality control standards for the components of Dalbergiae Odoriferae Lignum are still lacking. Consequently, there is a need to improve research in clinical pharmacology and pharmacodynamics within traditional Chinese medicine, identifying representative pharmacodynamic components and appropriate analytical methods to establish quality standards for Dalbergiae Odoriferae Lignum and its preparations [7].

Traditional Chinese medicine identification traditionally relies on sensory experiences, including visual observation of shape and colour, tactile examination of texture, olfaction of aroma, and taste assessment for the determination of medicinal material quality. This evaluation method is rooted in clinical experience but remains subjective and depends on individual expertise. The integration of modern bionic sensory technology can aid in quantifying and analysing medicinal materials and can scientifically explain the connection between appearance characteristics and quality [8,9,10]. By combining quantitative sensory indicators with active ingredients, the quality research and analysis of medicinal materials can be made more comprehensive. In recent years, several relevant studies have been conducted in this direction [11,12,13,14,15,16,17]. The 2020 edition of the Pharmacopoeia of the People’s Republic of China (referred to as the Chinese Pharmacopoeia) employs trait descriptions and thin-layer chromatography to authenticate Dalbergiae Odoriferae Lignum [1]. According to these standards, the ethanolic extract and volatile oil content must not be less than 8.0% and 1.0% (mL·g^−1^), respectively, to ensure quality control. However, this approach does not adequately assess the quality of Dalbergiae Odoriferae Lignum based solely on traits. Therefore, we sought to provide data to support the exploration of the relationship between traits and the quality of Dalbergiae Odoriferae Lignum. Among the observable traits, colour is the most intuitive and easily discernible, and colour variations often reflect the different qualities of medicinal materials [18]. Using biomimetic sensory technology, such as a colourimeter, to precisely quantify the colour of Dalbergiae Odoriferae Lignum and combining this with intrinsic chemical content, our aim was to validate whether colour traits indicate quality, ultimately establishing a swift and straightforward quality evaluation standard. The volatile oil of Dalbergiae Odoriferae Lignum is often used in practical applications, such as Qishen Yiqi Droplet, Guanxin Danshen Tablets, and other Chinese patent drugs. Hence, the specific constituents of the volatile oil were not determined to maintain the simplicity and practicability of the quality assessment. In this study, we used correlation, principal component analysis, and cluster analyses on colourimetric values (L*, a*, and b*) and the content of extract, volatile oil, total flavonoids, and six major flavonoid components found in Dalbergiae Odoriferae Lignum, with the objective of elucidating the key indicators that influence Dalbergiae Odoriferae Lignum quality and subsequently refining the quality evaluation method for this valuable medicinal material.

## 2. Results

### 2.1. Colouration

The chromaticity values of 60 batches of Dalbergiae Odoriferae Lignum were determined, and the L*, a*, and b* values of each batch of sample powder are presented in Table S1 (Supplementary Materials). A colourimeter can accurately quantify colours through the colour space of L*, a*, and b* by simulating the visual system of the human eye [19]. L*, a*, and b* represent the chromaticity value of the colour of an object, which specifically serves as the coordinates of the colour space of that colour, helping to locate the colour. Among these, L* signifies the brightness value, indicating the shade of the colour; it ranges from 0 to 100, where 0 corresponds to black and 100 to white. The a* axis depicts the red–green direction, with positive values indicating reddish tones and negative values representing green tones. Similarly, the b* axis signifies the yellow–blue direction, with positive values indicating yellowish hues and negative values indicating bluish hues.

### 2.2. Extract Content

The alcohol-soluble extract content of the 60 batches of Dalbergiae Odoriferae Lignum (Table S1) ranged from 12.17% to 42.74%. According to Pharmacopoeia guidelines, the extracted content of Dalbergiae Odoriferae Lignum should not be less than 8.0%; therefore, the extracted content of all samples was qualified.

### 2.3. Volatile Oil Content

The volatile oil contents of 60 batches of Dalbergiae Odoriferae Lignum (Table S1) ranged from 0.18% to 3.37%. The 2020 Chinese Pharmacopoeia mandates a minimum volatile oil content of 1.0% (mL·g^−1^); six batches (11, 12, 28, 31, 53, and 58) failed to meet this standard.

### 2.4. Total Flavonoids Content

The total flavonoids content of 60 batches of Dalbergiae Odoriferae Lignum (Table S1) ranged from 1.46% to 5.53% and varied substantially among the samples.

### 2.5. High-Performance Liquid Chromatography (HPLC) Determination of Six Flavonoids

The HPLC chromatograms of the six flavonoids in both the control and samples at the dual detection wavelengths of 275 and 350 nm are shown in Figure S1 (Supplementary Materials). The masses of the six flavonoids in Dalbergiae Odoriferae Lignum (liquiritigenin, butein, naringenin, isoliquiritigenin, formononetin, and pinocembrin) were calculated based on their respective peak areas; the results are summarised in Table S1.

### 2.6. Analysis of Variance (ANOVA) and Correlation Analysis

Among the 60 samples, six medicinal materials that did not meet the pharmacopoeial standards were excluded. The 12 indicators of the remaining 54 samples were analysed using descriptive statistics. The results are presented in Table 1. Pinocembrin, isoliquiritigenin, naringenin, butein, and formononetin showed the highest coefficients of variance, followed by b*, volatile oil, liquiritigenin, total flavonoids, extract, a*, and L*. Together, these results suggest that the larger the coefficient of variation, the greater the variability of the indicator. Accordingly, the 12 indicators, such as L*, a*, b*, extract, volatile oil, total flavonoids, and the contents of the six flavonoid compounds (liquiritigenin, butein, naringenin, isoliquiritigenin, formononetin, and pinocembrin), can be used as important indicators to classify the grades.

A correlation analysis was performed on the 12 indicators within the dataset of 54 samples to identify the key indicators for classifying medicinal materials. Based on the correlation matrix in Table 2, the colour parameter L* was highly and positively correlated with a* and b*. The higher the values of a* and b*, the higher the saturation and the more vivid the performance, which is consistent with the observation that the larger the value of L*, the lighter the colour. Among them, L* showed a strongly significant negative correlation with the content of the extract, volatile oil, total flavonoids, and naringenin. In addition, it demonstrated a significant negative correlation with the contents of formononetin and pinocembrin and a significant positive correlation with isoliquiritigenin content. L* represents the depth of the colour; the smaller the value of L*, the darker the colour, which implies that the darker the colour of Dalbergiae Odoriferae Lignum, the higher the content of the extract, volatile oil, total flavonoids, naringenin, formononetin, and pinocembrin, and the lower the content of isoliquiritigenin. Furthermore, a* showed a highly significant negative relationship with the extract and total flavonoids contents and a significant positive correlation with isoliquiritigenin content. The smaller the value of +a*, the darker the red colour; the darker the red colour, the greater the content of Dalbergiae Odoriferae Lignum extract and total flavonoids, and the smaller the content of isoliquiritigenin. Similarly, b* was significantly negatively correlated with the content of extract, volatile oil, total flavonoids, naringenin, and formononetin. Moreover, it was significantly negatively correlated with the pinocembrin content and significantly positively correlated with the isoliquiritigenin content. A higher +b* value corresponds to a yellowish–white colour, resembling the hue of sapwood. This suggested that the lighter yellow colour of Dalbergiae Odoriferae Lignum had lower quantities of extract, volatile oil, total flavonoids, naringenin, formononetin, and pinocembrin, whereas the isoliquiritigenin content was relatively higher. Liquiritigenin, isoliquiritigenin, and butein were not included as grading indicators in this study because of their notable correlations with a limited number of other indicators and their relatively low overall correlation coefficients.

### 2.7. Principal Component Analysis

ANOVA and correlation analysis yielded nine main indicators: extract, volatile oil, total flavonoids, naringenin, formononetin, pinocembrin, L*, a*, and b*, which were related to quality and were subjected to principal component analysis; the results are presented in Table 3. The proportion of the variance explained by each principal component varied. The first principal component eigenvalue was 5.095, and the proportion of variance explaining the original variable was 56.612. The second principal component eigenvalue was 1.578, and the proportion of variance explaining the original variable was 17.534. Two principal component factors with initial eigenvalues greater than 1 were extracted, indicating that these two principal components concentrated the most on the original indicators.

As shown in Table 4, the principal component matrix, extract, volatile oil, total flavonoids, naringenin, L*, and b* demonstrated high loading on the first principal component, implying that the first principal component could reflect the information carried by these indicators, whereas formononetin and a* exhibited high loadings on the second principal component, implying that the second principal component could reflect the information of these indicators. Comparing the contribution values of the nine indicators to the principal components, the indicators with larger vector values were extracted, volatile oil, total flavonoids, naringenin, L*, and b*, and the absolute values of the vector values of these six indicators were over 0.6; accordingly, these six indicators were primarily considered as the basis for classifying grades.

### 2.8. Hierarchical Cluster Analysis

**Figure 1:**
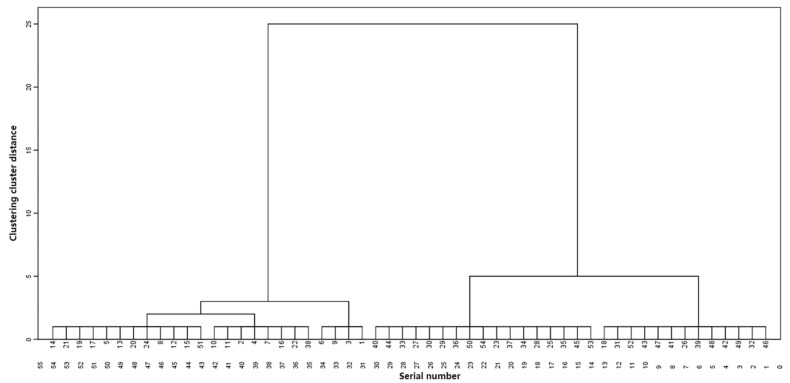
Hierarchical clustering dendrogram of Dalbergiae Odoriferae Lignum.

Through the examination of distinctions, correlations, and principal component analyses, various evaluation methods were considered. For the final comprehensive evaluation, six indicators, including extract, volatile oil, total flavonoids, naringenin, L*, and b*, were selected for hierarchical cluster analysis. A dendrogram was generated using Ward’s linkage method (Figure 1). The results revealed that the collected medicinal materials could be categorised into four major classes (i.e., four grades).

### 2.9. K-Means Cluster Analysis

ANOVA was employed to assess the contribution of the variables used for clustering to the clustering outcomes. A greater significance in the ANOVA test results indicated a stronger influence on the clustering results. The six indicators (extract, volatile oil, total flavonoids, naringenin, L*, and b*) were subjected to a K-cluster analysis. According to the ANOVA results (Table 5), the differences among the six indicators were found to be less than 0.01, signifying a high level of significance. The extract had the highest value of F, followed by L*, b*, and total flavonoids, indicating that these four indicators had a more important effect on the intrinsic quality of Dalbergiae Odoriferae Lignum and could be the main basis for the classification standard.

The results of the K-cluster analysis were categorised into four classes, similar to the hierarchical clustering results. The detailed classification centre values are listed in Table S2 (Supplementary Materials). Finally, six indices (extract, volatile oil, total flavonoids, naringenin, L*, and b*) were selected for the quality evaluation of Dalbergiae Odoriferae Lignum. By incorporating the cluster centres from the K-cluster analysis, this study formulated a quality grade standard for Dalbergiae Odoriferae Lignum, initially categorising it into four grades: first, second, third, and fourth. Based on the values of each cluster centre, the 2nd, 3rd, 4th, and 1st classes corresponded to the first, second, third, and fourth grades, respectively. In conjunction with the analysis of market prices, the pricing and classification outcomes for various grades of medicinal materials remained consistent, indicating that the evaluation criteria align with the market conditions (Supplementary Table S3).

## 3. Discussion

With the ever-increasing scarcity of resources and annual price hikes compounded by the absence of an accurate scientific method to verify the authenticity of Dalbergiae Odoriferae Lignum, the market has witnessed a surge in counterfeit and substandard products [20]. These counterfeits predominantly feature wood from tree species such as *Dalbergia oliveri* Gamble, *Dalbergia sissoo* Roxb., *Dalbergia hainanensis* Merr., and *Pterocarpus indicus* Willd [21,22]. Some tree species closely related to *D. odorifera* or similar in appearance are used to mimic genuine *D. odorifera* heartwood, often by colouring sapwood and infusing it with aromatic substances. These sub-par products frequently incorporate non-medicinal parts, including sapwood, wood processing remnants, extracts, volatile oils, and other materials that do not meet the quality standards. The current study conducted here plays a pivotal role in differentiating genuine Dalbergiae Odoriferae Lignum from a plethora of products in the market.

Traditionally and in modern times, the authenticity of medicinal materials can be determined through various techniques, including examination of appearance characteristics, microscopy, thin-layer chromatography, and DNA barcoding [23]. However, when evaluating the quality of stem wood medicinal materials such as Dalbergiae Odoriferae Lignum, reliance primarily on organoleptic indices remains the norm. Quantitative indices often concentrate on individual components, such as extracts and volatile oils, which may not comprehensively represent the overall quality or offer guidance for applications and production of traditional Chinese medicine. By conducting correlation and principal component analyses involving 12 key indicators, including colour, extract, volatile oil, and flavonoids, we identified extract, volatile oil, total flavonoids, naringenin, and chromaticity values (L* and b*) as crucial criteria for evaluating and grading the quality of Dalbergiae Odoriferae Lignum.

Among these, naringenin is a potentially significant medicinal ingredient in Dalbergiae Odoriferae Lignum, which warrants further investigation in future research. Dalbergiae Odoriferae Lignum exhibits higher levels of extract, volatile oil, total flavonoids, and naringenin, whereas those with a yellow and white colouration tend to have diminished concentrations of these components. As an initial observation, the darker shade of Dalbergiae Odoriferae Lignum typically signifies higher quality. By quantifying the colour of Dalbergiae Odoriferae Lignum powder and combining this with the intrinsic composition content, our study comprehensively analysed Dalbergiae Odoriferae Lignum. The ultimate objective was to establish a straightforward and standardised quality evaluation method for Dalbergiae Odoriferae Lignum, providing a scientific foundation for future development and use within the field of traditional Chinese medicine.

## 4. Materials and Methods

### 4.1. Instruments and Reagents

The following instruments and reagents were used: ME204E/02 electronic balance (Mettler-Toledo Instruments Co., Ltd., Shanghai, China), HunterLab UltraScan VIS Benchtop Spectrophotometer (Hunter Associates Laboratory, Inc., Reston, VA, USA), FW100 Portable High-Speed Universal Pulveriser (Tianjin Taiste Co., Ltd., Tianjin, China), Waters HPLC (including high-pressure binary pumps 1525, dual-wavelength detector 2487, and 717 autosampler) (Waters, Milford City, MA, USA), XSE105 one hundred thousandth balance (Mettler-Toledo Instruments Co., Ltd., Shanghai, China), KQ-500DE CNC Ultrasonic Cleaner (Kunshan Ultrasonic Instruments Co., Ltd., Kunshan, China), 95% ethanol and methanol (analytically pure), acetonitrile and methanol (chromatographically pure), and purified water (Wahaha Foods Co., Ltd., Hangzhou, China).

### 4.2. Standard Substances Selection

Although the main components of Dalbergiae Odoriferae Lignum are flavonoids and volatile oil, flavonoids are considered to be well-defined active ingredients in many medicinal plants. Therefore, flavonoids can be used as ‘marker compounds’ for the quality evaluation of Dalbergiae Odoriferae Lignum [24]. In Dalbergiae Odoriferae Lignum, there are approximately 99 flavonoids, of which more than 40 are considered active ingredients. Because of the requirement for cost-effective detection, we selected six major flavonoids as ‘marker compounds’. These were chosen because they constitute the majority of the total flavonoids and are active constituents of Dalbergiae Odoriferae Lignum. Liquiritigenin has anti-inflammatory and blood glucose-lowering activities; naringenin has anti-inflammatory and anti-oxidative activities; pinocembrin exhibits anti-inflammatory, anti-oxidative, anti-tumour, and vasodilatory activities; isoliquiritigenin has anti-inflammatory, blood glucose-lowering, and vasodilatory activities; formononetin has anti-oxidative, blood glucose-lowering, anti-tumour, and antibacterial activities [4,24,25,26,27,28,29,30,31]. The standard substances of the six flavonoids used in this study were liquiritigenin, naringenin, pinocembrin, butein, isoliquiritigenin, and formononetin. Information on the standard substances is presented in Table S4 (Supplementary Materials).

### 4.3. Sample Materials

Sixty samples were collected from Hainan, Guangdong, and other production areas. The samples were identified as *D. odorifera* heartwood by Associate Researcher Qi Yaodong at the Institute of Medicinal Plants of the Chinese Academy of Medical Sciences. All samples were free from moth and mildew infestations, and their properties were in accordance with the standards of the Chinese Pharmacopoeia. The retail price range is CNY 60–400/kg. Information on the samples is provided in Table S5 (Supplementary Materials).

### 4.4. Colour Test

Approximately 10 g of the sample powder, which had been crushed (40 mesh) and mixed well, were separately loaded into a quartz cuvette and placed on a HunterLab UltraScan VIS Benchtop Spectrophotometer (Hunter Associates Laboratory, Inc., Reston, VA, USA). The background was corrected, and measurements were conducted. Each sample was measured three times in parallel to obtain the average values.

Measurement conditions: We used the HunterLab UltraScan VIS Benchtop Spectrophotometer to detect the colour space L*, a*, and b* values of the colour quantitative indexes of the descending incense sample powder. A D65 projected light source was used with an SCE (Specular Component Exclude) reflection illumination system.

Data analysis method: EasyMatch QC 4.81 software (SensorManager Version 4.32; Colour Calculator Version 3.59) was used to collect colour data and transform parameters of the colour space L*, a*, and b*. Subsequently, the colour data were analysed and processed using IBM SPSS 20.0 software (IBM Corp., Armonk, NY, USA).

### 4.5. Determination of the Extract

Approximately 2 g of sample powder were added to 50 mL of 95% ethanol and weighed together. After heating under reflux for 1 h, the total weight of the extract was adjusted to its original weight using 95% ethanol. The obtained solution was filtered, and 25 mL of the filtrate were dried and subsequently accurately weighed. The extracted content in the test samples (%) was calculated. Each sample was measured three times in parallel to obtain the average values.

### 4.6. Determination of Volatile Oil

Approximately 50 g of the sample powder were taken, 500 mL of distilled water were added, and the mixture was heated for 6 h using the apparatus for determination of volatile oil (referred to the Chinese Pharmacopoeia Part IV on page 233). The volatile oil content in the test sample (%) was calculated. Each sample was measured three times in parallel to obtain the average values.

### 4.7. Determination of Total Flavonoids

#### 4.7.1. Preparation of the Test Solution

We precisely weighed 0.2000 g of Dalbergiae Odoriferae Lignum powder (40 mesh), pipetted 25 mL of 70% ethanol solution into a 100 mL conical flask, and placed it in a KQ-500DE CNC Ultrasonic Cleaner (Kunshan Ultrasonic Instruments Co., Ltd., Kunshan, China) (power 60 kHz, 25 °C) for 1 h. After extraction, the mixture was filtered, and the filtrate was fixed in a 70% ethanol solution in a 50 mL volumetric flask. Each sample was extracted thrice in parallel.

#### 4.7.2. Preparation of the Standard Curve

In this experiment, 2.33 mg of the naringenin control were precisely weighed using a 1 in 100,000 precision balance, dissolved in 70% ethanol, and volume adjusted to 25 mL. A total of 0.00, 0.50, 1.00, 1.50, 2.00, 2.50, and 3.00 mL of the control solution were pipetted into a 10 mL volumetric flask. Subsequently, 1 mL of sodium methoxide was added to the flask, and the volume was made up to 10 mL using 70% ethanol to 10 mL [26]. The mixture was then shaken and allowed to stand for 40 min, and the absorbance was measured at UV 410 nm. The regression equation was calculated as Y = 0.0209X − 0.0005, R^2^ = 0.998, and the linear range was 0.004–0.028 mg·mL^−1^. The standard curve is shown in Figure S2 (Supplementary Materials).

#### 4.7.3. Determination of the Test Solution

We added sodium methoxide to the test solution (0.5 mL) following the protocol outlined in Section 4.7.2. The solution was then analysed using a UV-spectrophotometer to determine its absorbance, and the average value was recorded. This value was then used to calculate the total flavonoids content using the standard curve equation described in Section 4.7.2.

### 4.8. HPLC Determination of Six Flavonoids in Dalbergiae Odoriferae Lignum

#### 4.8.1. Preparation of the Test Solution

We accurately weighed 0.1000 g of Dalbergiae Odoriferae Lignum powder (40 mesh) and placed it in a conical flask. Subsequently, 10 mL of 60% methanol solution were pipetted, weighed, and subjected to ultrasonic extraction for 1 h (power, 60 kHz; temperature, 25 °C). After cooling, the weight loss was compensated with 60% methanol. The extracted solution was filtered through a 0.22 µm microporous filter membrane in a sample bottle, and the test solution was obtained. The test solution was refrigerated at 4 °C prior to the assay.

#### 4.8.2. Preparation of the Control Solution

Appropriate amounts of liquiritigenin, naringenin, pinocembrin, butein, isoliquiritigenin, and formononetin were accurately measured, dissolved, and combined with methanol in 5 mL volumetric flasks. A volume of 1 mL of each of the single standard master batch solutions was pipetted and diluted with methanol to reach a total volume of 10 mL. The mixture was thoroughly mixed to obtain the standard solution. Subsequently, the solution was stored at −20 °C for preservation. Before sampling, the solution was filtered using a 0.22 µm microporous membrane.

#### 4.8.3. Chromatographic Conditions

Waters HPLC (including high-pressure binary pumps 1525, dual-wavelength detector 2487, and 717 autosampler) (Waters, Milford City, MA, USA) with Agilent ZORBAX SB-C_18_ column (250 mm × 4.6 mm, 5 µm) was used to determine the contents of the six flavonoids, with a flow rate of 0.8 mL·min^−1^. The dual detection wavelengths were performed at 275 nm and 350 nm for wavelengths 1 and 2, respectively. The injection volume was 10 µL, and the mobile phases were acetonitrile (A) and 0.3% acetic acid (B) [24,32]. The gradient elution conditions were as follows: 0–16 min, 26% A, and 16–65 min, 26%–40% A.

### 4.9. Data Processing and Classification Methods

All L*, a*, b*, extract, volatile oil, total flavonoids, and flavonoids assay data were imported into SPSS 20.0 for correlation analysis. These indicators were individually clustered and analysed to generate a cluster dendrogram. Grade classification of Dalbergiae Odoriferae Lignum was conducted based on the clustering results.

## 5. Conclusions

Given the limited time frame, this study focused solely on analysing these six flavonoids. Future research must expand the scope of the analysis to encompass a more diverse range of compounds. This study mainly focused on colour traits; the analysis could be enhanced using tools such as electronic noses and electronic tongues to assess the aroma and taste properties of Dalbergiae Odoriferae Lignum. Further studies should consider in-depth analyses of volatile components and other traits that reflect quality, thus enhancing the quality evaluation system for Dalbergiae Odoriferae Lignum. This approach provides a more robust reference for establishing quality standards and classifying the grades of perennial woody plants.

## Figures and Tables

**Table 1:** Description of data on 12 indicators for 54 batches of qualified samples (*n* = 54).

Indicator	Minimum Value	Maximum Value	Average Value	Standard Deviation	Variance	Coefficient of Variation
Extract/%	14.81	42.74	27.38	6.92	47.9	25.28
Volatile oil/%	1.05	3.37	1.92	0.59	0.35	30.68
Total flavonoids/%	1.46	5.53	3.67	0.93	0.87	25.42
Liquiritigenin/%	0.06	0.55	0.27	0.08	0.01	28.58
Naringenin/%	0.16	1.97	0.73	0.36	0.13	49.41
Formononetin/%	0.02	0.16	0.07	0.03	0.00	46.26
Pinocembrin/%	0.01	0.29	0.09	0.06	0.00	63.39
Isoliquiritigenin/%	0.00	0.54	0.13	0.07	0.00	52.25
Butein/%	0.00	0.42	0.15	0.07	0.01	47.37
L*	38.98	52.91	44.59	3.29	10.80	7.37
a*	5.93	12.84	9.71	1.65	2.72	16.98
b*	3.83	16.88	9.44	2.93	8.61	31.09

**Table 2:** Correlation analysis of 12 indicators in 54 batches of qualified samples.

Indicator	Extract	Volatile Oil	Total Flavonoids	Liquiritigenin	Naringenin	Formononetin	Pinocembrin	Isoliquiritigenin	Butein	L*	a*
Volatile oil	0.661 **										
Total flavonoids	0.800 **	0.502 **									
Liquiritigenin	0.158	0.168	0.342 *								
Naringenin	0.522 **	0.476 **	0.505 **	0.390 **							
Formononetin	0.562 **	0.333 *	0.402 **	0.254	0.232						
Pinocembrin	0.477 **	0.403 **	0.408 **	0.261	0.656 **	0.407 **					
Isoliquiritigenin	−0.239	−0.290 *	0.105	0.682 **	−0.075	0.009	−0.059				
Butein	−0.150	0.011	−0.057	0.262	0.391 **	−0.410 **	0.301 *	0.032			
L*	−0.812 **	−0.640 **	-0.754 **	−0.065	−0.486 **	−0.302 *	−0.340 *	0.321 *	0.077		
a*	−0.457 **	−0.241	−0.497 **	0.087	−0.216	0.145	0.020	0.318 *	−0.072	0.694 **	
b*	−0.817 **	−0.533 **	−0.777 **	0.024	−0.369 **	−0.333 **	−0.285 *	0.297 *	0.204	0.958 **	0.746 **

Note: * and ** indicate a significant correlation at *p* < 0.05 level and a highly significant correlation at *p* < 0.01 level, respectively.

**Table 3:** Total interpretation of variance for the extracted principal components.

Principal Component	Initial Eigenvalue			Extract the Sum of Squares and Load		
	Summation	Variance Contribution/%	Cumulative Contribution/%	Summation	Variance Contribution/%	Cumulative Contribution/%
1	5.095	56.612	56.612	5.095	56.612	56.612
2	1.578	17.534	74.146	1.578	17.534	74.146
3	0.887	9.851	83.996			
4	0.563	6.261	90.257			
5	0.314	3.491	93.748			
6	0.267	2.971	96.719			
7	0.147	1.634	98.353			
8	0.129	1.428	99.781			
9	0.020	0.219	100.000			

**Table 4:** Component matrix of the two principal components.

Principal Component	Extract	Volatile Oil	Total Flavonoids	Naringenin	Formononetin	Pinocembrin	L*	a*	b*
1	0.927	0.721	0.864	0.647	0.483	0.554	−0.924	−0.586	−0.902
2	0.064	0.166	−0.055	0.366	0.551	0.629	0.270	0.720	0.345

**Table 5:** Analysis of variance (ANOVA) results.

Indicator	Clustering		Inaccuracies			
	Mean Square	df	Mean Square	df	F	Sig.
Extract	744.925	3	4.685	49	158.989	0.000
Volatile oil	2.980	3	0.187	49	15.927	0.000
Total flavonoids	10.202	3	0.293	49	34.835	0.000
Naringenin	0.625	3	0.104	49	6.037	0.001
L*	147.018	3	2.404	49	61.144	0.000
b*	115.068	3	1.843	49	62.423	0.000

## Data Availability

Data are contained within the article and supplementary materials.

## Supplementary Materials

The following supporting information can be downloaded at: https://www.mdpi.com/article/10.3390/molecules28227635/s1, Figure S1. HPLC chromatograms of six flavonoid standards and samples about Dalbergiae Odoriferae Lignum; Figure S2. Standard curve of the absorbance value (X) and concentration (Y) of naringenin; Table S1. Data of the 60 batches of Dalbergiae Odoriferae Lignum (X ± SD, *n* = 3); Table S2. Clustering centres of grading indicators for Dalbergiae Odoriferae Lignum; Table S3. Quality grading of Dalbergiae Odoriferae Lignum; Table S4. Chemical information and sources of the six standard substances; Table S5. Source, identification and retail price information of 60 batch samples.
