# Supplementary material for: Correlation between Colour Traits and Intrinsic Quality of Dalbergiae Odoriferae Lignum
Source: Molecules. 2023 Nov 16;28(22):7635. doi: 10.3390/molecules28227635 (PMC10674628; doi:10.3390/molecules28227635)
Supplement: Supplementary file 1 [file molecules-28-07635-s001.zip › molecules-2635727-supplementary.pdf]

molecules

Supplementary Materials

Correlation between Colour Traits and Intrinsic Quality of Dalbergiae Odoriferae Lignum

Wenjie He [1,2,†], Ying Sun [3,†], Sai Zhang [1,2], Jiawen Li [2], Jixing Feng [2], Yun Yang [2], Hui Meng [2,*] and Zheng Zhang [1,*]

[1] Institute of Medicinal Plant Development, Chinese Academy of Medical Sciences and Peking Union Medical College, Beijing 100193, China
[2] Key Laboratory of Resources Conservation and Development of Southern Medicine of Hainan Province & Hainan Branch of the Institute of Medicinal Plant Development, Chinese Academy of Medical Sciences and Peking Union Medical College, Haikou 570311, China
[3] Hainan Hospital of Chinese PLA General Hospital, Sanya 572000, China
* Correspondence: huiziqq@163.com (H.M.); zhangzheng@implad.ac.cn (Z.Z.); Tel.: +86-898-3158-9019 (H.M.)
† These authors contributed equally to this work.

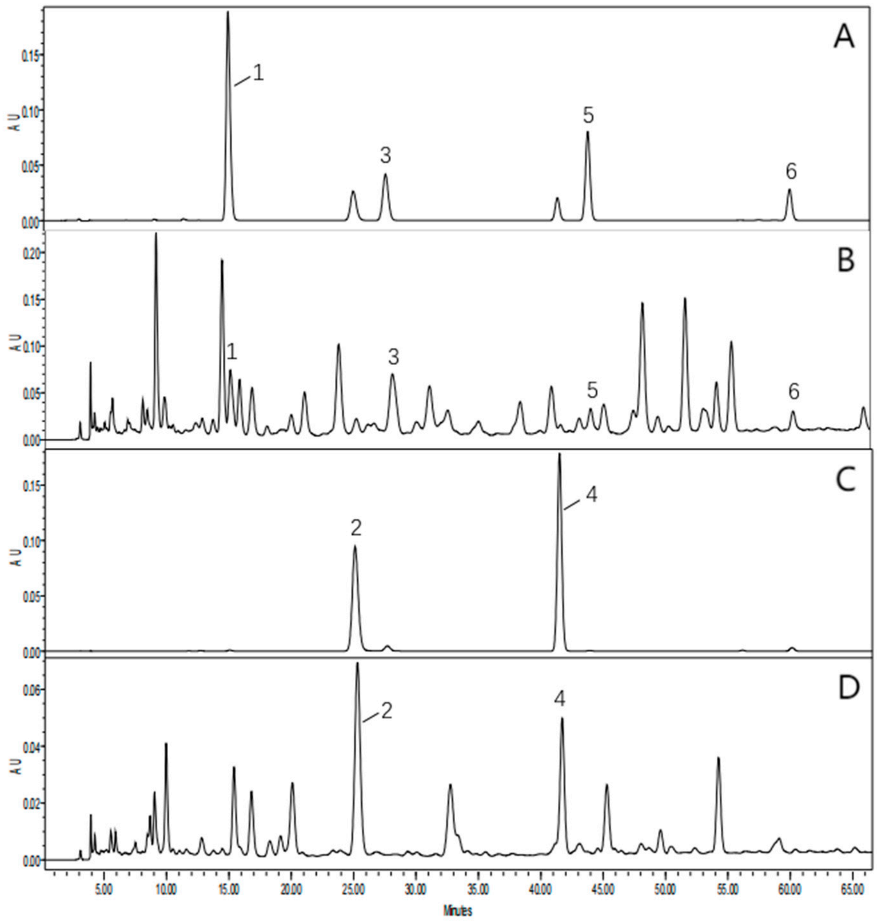

Figure S1. HPLC chromatograms of six flavonoid standards and samples about Dalbergiae Odoriferae Lignum.

Note: **A**. Control solution (275 nm), **B**. Test solution (275 nm), **C**. Control solution (350 nm), **D**. Test solution (350 nm); peaks 1–6 represent liquiritigenin, butein, naringenin, isoliquiritigenin, formononetin, and pinocembrin.

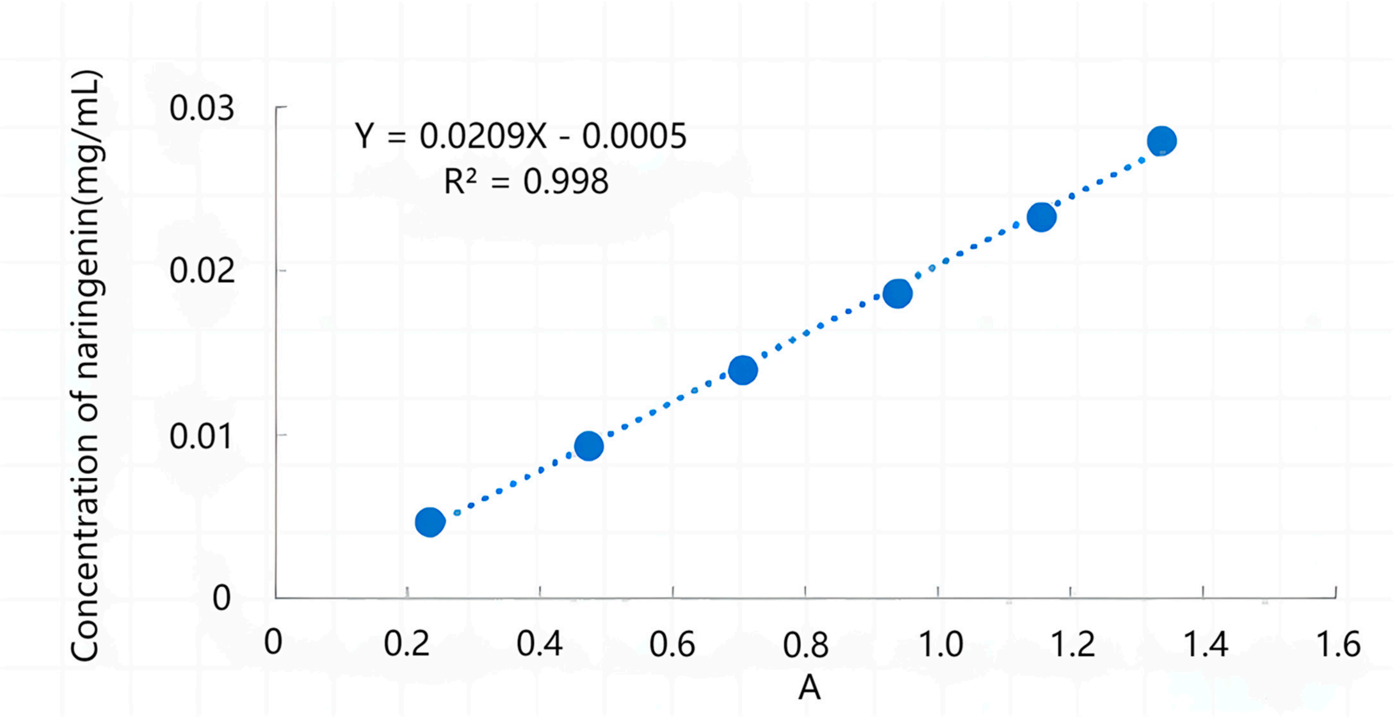

Figure S2. Standard curve of the absorbance value (X) and concentration (Y) of naringenin.

molecules MDPI

Table S1. Data of the 60 batches of Dalbergiae Odoriferae Lignum (X ± SD, *n* = 3).

Serial number	Extract%±SD%	Volatile oil%±SD%	Total flavonoids%±SD%	Liquiritigenin%±SD%	Naringenin%±SD%	Formononetin%±SD%	Pinocembrin%±SD%	Isoliquiritigenin%±SD%	Butein%±SD%	*L**	*a**	*b**
1	26.65±0.0011	1.81±0.0018	3.20±0.006	0.22±0.0001	0.68±0.0004	0.05±0.0000	0.10±0.0000	0.11±0.0000	0.13±0.0007	43.80±0.2627	9.85±0.4466	8.52±0.5008
2	30.83±0.0047	2.24±0.0034	3.92±0.0005	0.31±0.0002	0.82±0.0004	0.11±0.0001	0.11±0.0002	0.12±0.0001	0.13±0.0007	43.99±0.1960	10.01±0.1030	8.74±0.1562
3	34.39±0.0029	2.02±0.0039	3.75±0.0010	0.32±0.0001	0.78±0.0002	0.06±0.0000	0.08±0.0000	0.10±0.0000	0.16±0.0003	44.42±0.2479	10.00±0.1361	9.61±0.0416
4	29.29±0.0017	1.74±0.0047	3.17±0.0007	0.24±0.0001	0.67±0.0004	0.10±0.0000	0.08±0.0000	0.10±0.0000	0.11±0.0001	45.11±0.2259	10.94±0.2548	9.64±0.3717
5	31.44±0.0004	2.63±0.0007	4.32±0.0005	0.22±0.0001	0.67±0.0003	0.05±0.0000	0.09±0.0007	0.11±0.0001	0.20±0.0001	40.48±0.0929	6.72±0.0289	4.69±0.0231
6	24.01±0.0017	1.65±0.0004	3.56±0.0003	0.34±0.0000	1.31±0.0001	0.06±0.0000	0.18±0.0000	0.15±0.0000	0.23±0.0001	46.81±0.1752	11.75±0.1700	11.31±0.2193
7	22.98±0.0018	1.71±0.0011	3.34±0.0003	0.29±0.0003	0.59±0.0006	0.07±0.0001	0.05±0.0001	0.15±0.0001	0.12±0.0002	47.33±0.1217	11.45±0.0569	11.86±0.0709
8	30.28±0.0039	2.70±1.0010	3.35±0.0003	0.34±0.0001	1.07±0.0005	0.02±0.0000	0.10±0.0001	0.14±0.0001	0.33±0.0001	44.32±0.1947	10.38±0.0802	10.81±0.1358
9	24.62±0.0012	1.70±0.0005	3.13±0.0001	0.28±0.0001	0.57±0.0002	0.08±0.0000	0.08±0.0000	0.14±0.0000	0.19±0.0001	46.44±0.3066	10.92±0.1044	10.79±0.3200
10	27.80±0.0025	2.18±0.0017	3.31±0.0007	0.23±0.0001	0.47±0.0004	0.06±0.0000	0.20±0.0002	0.09±0.0001	0.15±0.0001	43.94±0.0950	10.29±0.0723	8.95±0.0751
11	12.17±0.0024	0.83±0.0005	1.56±0.0001	0.16±0.0001	0.27±0.0002	0.04±0.0000	0.03±0.0000	0.12±0.0001	0.10±0.0002	53.17±0.0889	11.43±0.0954	15.59±0.1044
12	12.29±0.0012	0.78±0.0002	1.78±0.0008	0.25±0.0001	0.30±0.0001	0.05±0.0000	0.04±0.0000	0.11±0.0000	0.13±0.0000	51.52±0.2910	10.99±0.0700	14.83±0.1249
13	17.06±0.0031	1.06±0.0011	2.43±0.0004	0.23±0.0001	0.45±0.0002	0.02±0.0000	0.03±0.0000	0.10±0.0001	0.17±0.0000	48.28±0.1823	8.15±0.0557	11.95±0.2100
14	14.81±0.0012	1.14±0.0009	1.80±0.0004	0.18±0.0001	0.33±0.0001	0.05±0.0000	0.05±0.0000	0.11±0.0001	0.13±0.0001	51.55±0.1387	11.07±0.0208	14.26±0.0929

15	19.26±0.0008	1.63±0.0012	3.32±0.0006	0.28±0.0001	0.65±0.0003	0.02±0.0000	0.07±0.0000	0.16±0.0001	0.28±0.0000	46.33±0.1258	8.32±0.0702	11.30±0.1050
16	35.72±0.0013	2.30±0.0042	4.67±0.0005	0.27±0.0002	0.88±0.0007	0.12±0.0001	0.17±0.0002	0.08±0.0001	0.13±0.0000	41.62±0.3066	7.92±0.1790	6.05±0.1418
17	22.50±0.0004	1.54±0.0023	2.88±0.0003	0.06±0.0001	0.33±0.0006	0.04±0.0001	0.01±0.0001	0.00±0.0000	0.00±0.0001	42.58±0.0757	8.37±0.0833	7.22±0.1800
18	42.74±0.0006	2.51±0.0075	5.26±0.0008	0.19±0.0004	1.11±0.0021	0.06±0.0001	0.11±0.0004	0.05±0.0001	0.11±0.0001	38.98±0.0586	6.06±0.0700	3.96±0.070
19	30.78±0.0017	1.82±0.0006	4.12±0.0002	0.32±0.0001	0.73±0.0002	0.08±0.0000	0.16±0.0000	0.14±0.0000	0.09±0.0000	42.61±0.0833	9.33±0.1365	7.41±0.1552
20	35.06±0.0011	1.88±0.0019	5.05±0.0009	0.22±0.0001	0.82±0.0003	0.05±0.0000	0.10±0.0000	0.08±0.0000	0.18±0.0001	39.78±0.0321	5.93±0.0351	3.82±0.0700
21	37.15±0.0011	2.67±0.0022	4.20±0.0010	0.34±0.0001	1.46±0.0011	0.09±0.0001	0.25±0.0004	0.13±0.0000	0.19±0.0002	43.96±0.0964	10.30±0.1026	9.41±0.0889
22	30.65±0.0032	1.85±0.0014	3.85±0.0007	0.18±0.0002	0.27±0.0002	0.05±0.0000	0.02±0.0000	0.09±0.0001	0.12±0.0006	45.63±0.1222	10.49±0.0451	10.45±0.0755
23	32.17±0.0041	2.29±0.0037	3.24±0.0005	0.37±0.0001	0.79±0.0003	0.10±0.0000	0.13±0.0001	0.11±0.0000	0.12±0.0000	44.15±0.1778	9.82±0.1484	9.28±0.1955
24	32.69±0.0020	2.42±0.002	4.18±0.0010	0.31±0.0000	1.12±0.0001	0.07±0.0000	0.15±0.0000	0.13±0.0000	0.22±0.0001	43.92±0.1286	10.89±0.1159	9.94±0.1473
25	30.88±0.0060	1.88±0.0003	4.28±0.0010	0.19±0.0004	0.43±0.0008	0.04±0.0001	0.01±0.0000	0.09±0.0002	0.15±0.0003	42.49±0.1940	8.56±0.1400	6.81±0.1422
26	27.98±0.0018	2.45±0.0034	4.26±0.0008	0.32±0.0002	1.17±0.0012	0.05±0.0000	0.16±0.0001	0.14±0.0001	0.24±0.0001	42.02±0.1609	9.62±0.0600	8.80±0.1997
27	17.88±0.0023	1.32±0.0009	2.56±0.0004	0.32±0.0015	0.43±0.0004	0.06±0.0006	0.06±0.0005	0.17±0.0011	0.21±0.0002	48.22±0.1701	10.29±0.1250	12.82±0.2914
28	24.93±0.0022	0.45±0.0006	3.97±0.0005	0.41±0.0001	0.18±0.0000	0.10±0.0000	0.04±0.0000	0.16±0.0001	0.03±0.0013	48.49±0.3381	12.93±0.1929	12.33±0.2822
29	19.39±0.0014	1.73±0.0022	3.68±0.0007	0.37±0.0001	1.07±0.0003	0.02±0.0000	0.17±0.0001	0.14±0.0000	0.42±0.0000	43.52±0.0839	8.86±0.1758	9.65±0.2957
30	25.60±0.0010	1.77±0.0020	2.79±0.0005	0.22±0.0000	0.71±0.0002	0.10±0.0000	0.01±0.0000	0.12±0.0000	0.07±0.0006	46.51±0.1026	12.67±0.0723	11.38±0.1345
31	17.46±0.0017	0.95±0.0017	2.26±0.0005	0.24±0.0000	0.31±0.0001	0.07±0.0000	0.04±0.0000	0.17±0.0000	0.30±0.0003	51.15±0.0656	9.59±0.0265	14.18±0.1193

32	23.81±0.0019	2.96±0.0216	3.74±0.0006	0.37±0.0001	0.77±0.0003	0.10±0.0001	0.08±0.0000	0.11±0.0001	0.15±0.0000	43.23±0.1531	11.31±0.1550	10.95±0.1852
33	18.84±0.0023	1.33±0.0015	2.64±0.0005	0.30±0.0001	0.59±0.0004	0.03±0.0001	0.03±0.0000	0.14±0.0001	0.22±0.0001	48.25±0.2346	9.54±0.5034	13.97±0.8544
34	19.51±0.0022	1.08±0.0011	2.53±0.004	0.25±0.0001	0.75±0.0004	0.04±0.0000	0.05±0.0000	0.16±0.0001	0.10±0.0002	46.55±0.1210	9.20±0.0404	11.08±0.0656
35	19.78±0.0043	1.15±0.0012	3.77±0.0004	0.31±0.0007	0.89±0.0041	0.03±0.0001	0.07±0.0000	0.19±0.0005	0.27±0.0001	46.44±0.1012	9.51±0.1804	12.14±0.4255
36	33.95±0.0018	1.82±0.0031	4.69±0.0010	0.29±0.0000	0.82±0.0007	0.07±0.0000	0.09±0.0001	0.12±0.0000	0.17±0.0000	39.95±0.0306	8.13±0.0833	5.49±0.0902
37	34.67±0.0022	3.06±0.0044	4.37±0.0005	0.24±0.0001	0.65±0.0003	0.06±0.0000	0.07±0.0004	0.08±0.0000	0.11±0.0000	39.10±0.0985	6.68±0.0252	4.77±0.0379
38	34.03±0.0026	2.29±0.0027	4.36±0.0013	0.40±0.0001	0.87±0.0001	0.12±0.0000	0.09±0.0003	0.14±0.0000	0.09±0.0000	41.21±0.0252	7.86±0.1626	6.22±0.1012
39	35.97±0.0039	1.67±0.0011	5.53±0.0006	0.31±0.0001	1.65±0.0003	0.08±0.0000	0.11±0.0001	0.13±0.0000	0.22±0.0001	39.24±0.1280	6.52±0.0808	4.51±0.0557
40	36.81±0.0042	2.33±0.0023	4.65±0.0017	0.21±0.0018	1.35±0.0001	0.15±0.0000	0.29±0.0000	0.12±0.0000	0.13±0.0000	43.04±0.0872	12.84±0.0426	8.45±0.0200
41	29.95±0.0012	3.37±0.0029	4.74±0.0012	0.24±0.0000	0.44±0.0000	0.09±0.0000	0.07±0.0000	0.08±0.0000	0.08±0.0000	43.88±0.0473	10.36±0.0493	8.68±0.0351
42	22.26±0.0027	1.21±0.0013	3.40±0.0004	0.21±0.0002	0.49±0.0003	0.06±0.0000	0.08±0.0001	0.10±0.0001	0.10±0.0001	44.07±0.0351	10.21±0.0624	9.01±0.0764
43	28.19±0.0037	2.02±0.0002	3.62±0.0006	0.29±0.0001	0.71±0.0001	0.08±0.0000	0.11±0.0000	0.15±0.0000	0.15±0.0000	45.16±0.0611	11.07±0.1358	9.85±0.2122
44	29.72±0.0012	2.26±0.0023	3.68±0.0004	0.28±0.0001	0.74±0.0003	0.08±0.0000	0.11±0.0001	0.14±0.0001	0.15±0.0001	45.40±0.0306	10.88±0.0681	9.52±0.0569
45	30.82±0.0062	1.87±0.0011	3.60±0.0010	0.29±0.0001	0.67±0.0002	0.07±0.0000	0.05±0.0000	0.09±0.0001	0.13±0.0000	43.36±0.0416	9.72±0.0252	9.13±0.0723
46	21.93±0.0018	1.09±0.0004	4.95±0.0007	0.55±0.0009	0.16±0.0011	0.06±0.0001	0.03±0.0000	0.54±0.0009	0.04±0.0001	48.68±0.1692	11.81±0.0800	11.85±0.0945
47	22.91±0.0017	1.53±0.0024	3.47±0.0004	0.26±0.0005	0.47±0.0011	0.07±0.0001	0.08±0.0002	0.15±0.0003	0.11±0.0002	47.54±0.1301	11.15±0.0306	11.43±0.0681
48	22.40±0.0041	1.70±0.0016	2.95±0.0011	0.29±0.0002	0.72±0.0006	0.07±0.0000	0.07±0.0000	0.16±0.0004	0.14±0.0001	47.15±0.0808	11.10±0.0100	11.36±0.0361

49	25.28±0.0012	1.38±0.0005	3.37±0.0004	0.26±0.0002	0.61±0.0005	0.07±0.0000	0.07±0.0000	0.16±0.0002	0.15±0.0001	45.77±0.0902	10.23±0.0929	10.06±0.1493
50	29.74±0.0045	3.33±0.0021	4.10±0.0013	0.40±0.0001	1.97±0.0007	0.04±0.0004	0.11±0.0003	0.13±0.0000	0.19 ±0.0001	42.49±0.2702	9.16±0.1665	9.31±0.2710
51	18.51±0.0010	1.05±0.0011	1.70±0.0010	0.22±0.0001	0.28±0.0001	0.03±0.0000	0.07±0.0000	0.09±0.0001	0.26±0.0002	52.91±0.2250	11.28±0.1429	16.88±0.1345
52	15.33±0.0070	1.07±0.0008	2.98±0.0009	0.20±0.0001	0.32±0.00003	0.05±0.0000	0.04±0.0000	0.15±0.0001	0.15±0.0000	52.63±0.1908	11.47±0.0737	15.18±0.1680
53	16.53±0.0034	0.99±0.0004	1.37±0.0009	0.15±0.0002	0.28±0.0005	0.04±0.0000	0.04±0.0001	0.10±0.0001	0.13±0.0002	52.07±0.1054	11.20±0.0346	14.45±0.0208
54	15.14±0.0068	1.23±0.0020	1.94±0.0005	0.17±0.0001	0.33±0.0002	0.04±0.0000	0.04±0.0000	0.11±0.0001	0.09±0.0001	51.12±0.3724	11.60±0.0231	14.49±0.3182
55	20.84±0.0014	2.27±0.0074	1.46±0.0001	0.18±0.0001	0.16±0.00003	0.05±0.0000	0.02±0.0000	0.10±0.0000	0.13±0.0001	44.67±0.1221	10.05±0.0361	10.30±0.1153
56	34.95±0.0021	2.51±0.0012	4.94±0.0003	0.27±0.0001	0.62±0.0002	0.07±0.0000	0.08±0.0000	0.09±0.0000	0.08±0.0000	41.11±0.0600	7.72±0.0586	5.59±0.0473
57	35.59±0.0021	2.37±0.0032	4.97±0.0004	0.23±0.0001	0.93±0.0005	0.07±0.0001	0.12±0.0001	0.10±0.0001	0.13±0.0001	40.67±0.0404	7.25±0.0265	4.93±0.0321
58	16.53±0.0003	0.18±0.0003	2.90±0.0008	0.19±0.0000	0.03±0.0000	0.02±0.0000	0.00±0.0000	0.10±0.0000	0.00±0.0000	43.51±0.3843	9.60±0.2476	8.97±0.2854
59	23.12±0.0025	1.38±0.0003	3.36±0.0003	0.21±0.0001	0.43±0.0001	0.04±0.0001	0.04±0.0000	0.10±0.0000	0.06±0.0000	43.40±0.2802	10.04±0.3745	8.82±0.4027
60	39.60±0.0040	1.93±0.0010	4.85±0.0003	0.42±0.0001	0.87±0.0002	0.16±0.0000	0.10±0.0003	0.15±0.0001	0.10±0.0001	41.84±0.0416	8.59±0.0458	6.31±0.0379

Table S2. Clustering centres of grading indicators for Dalbergiae Odoriferae Lignum.

Form	Extract/%	Volatile oil/%	Total flavonoids/%	Naringenin/%	*L**	*b**
Category 1	16.46	1.15	2.24	0.36	50.79	14.26
Category 2	35.98	2.31	4.76	0.98	40.84	5.71
Category 3	30.17	2.25	3.79	0.80	43.93	9.15
Category 4	22.36	1.62	3.28	0.63	45.71	10.62

Table S3. Quality grading of Dalbergiae Odoriferae Lignum.

Serial number	Source of product	Retail Price(CN¥)[a]	Extract/%	Volatile oil/%	Total flavonoids/%	Naringenin/%	*L**	*b**	Hierarchy
5	Xinyi	350	31.44	2.63	4.32	0.67	40.48	4.69	first grade
16	Huazhou	350	35.72	2.30	4.67	0.88	41.62	6.05	first grade
18	Qingyuan	350	42.74	2.51	5.26	1.11	38.98	3.96	first grade
37	Haikou	400	34.67	3.06	4.37	0.65	39.10	4.77	first grade
38	Qingyuan	350	34.03	2.29	4.36	0.87	41.21	6.22	first grade
40	Xinyi	350	36.81	2.33	4.65	1.35	43.04	8.45	first grade
56	Xinyi	350	34.95	2.51	4.94	0.62	41.11	5.59	first grade
57	Huazhou	350	35.59	2.37	4.97	0.93	40.67	4.93	first grade
2	Haikou	260	30.83	2.24	3.92	0.82	43.99	8.74	second grade
3	Qingyuan	300	34.39	2.02	3.75	0.78	44.42	9.61	second grade
21	Huazhou	320	37.15	2.67	4.20	1.46	43.96	9.41	second grade
22	Haikou	260	30.65	1.85	3.85	0.27	45.63	10.45	second grade
23	Qingyuan	300	32.17	2.29	3.24	0.79	44.15	9.28	second grade
24	Wanning	260	32.69	2.42	4.18	1.12	43.92	9.94	second grade
25	Xinyi	300	30.88	1.88	4.28	0.43	42.55	6.86	second grade
41	Xinyi	350	29.95	3.37	4.74	0.44	43.88	8.68	second grade
43	Haikou	260	28.19	2.02	3.62	0.71	45.16	9.85	second grade
44	Wanning	260	29.72	2.26	3.68	0.74	45.40	9.52	second grade
45	Qingyuan	300	30.82	1.87	3.60	0.67	43.36	9.13	second grade
19	Wanning	260	30.78	1.82	4.12	0.73	42.61	7.41	second grade
20	Xinyi	300	35.06	1.88	5.05	0.82	39.81	3.83	second grade
36	Huazhou	320	33.95	1.82	4.69	0.82	39.92	5.50	second grade
39	Wanning	260	35.97	1.67	5.53	1.65	39.24	4.51	second grade
60	Qingyuan	300	39.60	1.93	4.85	0.87	41.84	6.31	second grade
10	Xinyi	350	27.80	2.18	3.31	0.47	43.94	8.95	second grade
26	Huazhou	320	27.98	2.45	4.26	1.17	42.02	8.80	second grade
50	Qingyuan	300	29.74	3.33	4.10	1.97	42.49	9.31	second grade
1	Huazhou	280	26.65	1.81	3.20	0.68	43.80	8.52	third grade
4	Wanning	260	29.29	1.74	3.17	0.67	45.11	9.64	third grade
42	Huazhou	200	22.26	1.21	3.40	0.49	44.07	9.01	third grade

6	Huazhou	200	24.01	1.65	3.56	1.31	46.81	11.31	third grade
7	Haikou	200	22.98	1.71	3.34	0.59	47.33	11.86	third grade
8	Qingyuan	300	30.28	2.70	3.35	1.07	44.32	10.81	third grade
9	Wanning	260	24.62	1.70	3.13	0.57	46.44	10.79	third grade
27	Haikou	180	17.88	1.32	2.56	0.43	48.22	12.82	third grade
29	Wanning	260	19.39	1.73	3.68	1.07	43.52	9.65	third grade
30	Xinyi	200	25.60	1.77	2.79	0.71	46.51	11.38	third grade
47	Huazhou	200	22.91	1.53	3.47	0.47	47.54	11.43	third grade
48	Haikou	200	22.40	1.70	2.95	0.72	47.15	11.36	third grade
49	Wanning	260	25.28	1.38	3.37	0.61	45.77	10.06	third grade
17	Haikou	200	22.50	1.54	2.88	0.33	42.58	7.22	third grade
59	Wanning	260	23.12	1.38	3.36	0.43	43.40	8.82	third grade
32	Haikou	200	23.81	2.96	3.74	0.77	43.23	10.95	third grade
33	Qingyuan	200	18.84	1.33	2.64	0.59	48.25	13.97	third grade
46	Xinyi	200	21.93	1.09	4.95	0.16	48.68	11.85	fourth grade
13	Qingyuan	80	17.06	1.06	2.43	0.45	48.28	11.95	fourth grade
14	Wanning	200	14.81	1.14	1.80	0.33	51.55	14.26	fourth grade
15	Xinyi	200	19.26	1.63	3.32	0.65	46.33	11.30	fourth grade
34	Wanning	200	19.51	1.08	2.53	0.75	46.55	11.08	fourth grade
35	Xinyi	200	19.78	1.15	3.77	0.89	46.44	12.14	fourth grade
51	Xinyi	200	18.51	1.05	1.70	0.28	52.91	16.88	fourth grade
52	Huazhou	100	15.33	1.07	2.98	0.32	52.63	15.18	fourth grade
54	Wanning	200	15.14	1.23	1.94	0.33	51.12	14.49	fourth grade
55	Qingyuan	200	20.84	2.27	1.46	0.16	44.67	10.30	fourth grade

Note:[a]The retail price corresponds to 1 kg.

Table S4. Chemical information and sources of the six standard substances.

Standard substances	CAS no.	Molecular formula (chemistry)	MW	Flavonoid type	Source of standard substances
Liquiritigenin	578-86-9	$C_{15}H_{12}O_4$	256	Flavanones	Sigma-Aldrich(Shanghai) Trading Co., Ltd., Shanghai, China
Naringenin	480-14-1	$C_{15}H_{12}O_5$	272	Flavanones	Shanghai Aladdin Biochemical Technology Co., Ltd., Shanghai, China
Pinocembrin	480-39-7	$C_{15}H_{12}O_4$	256	Flavanones	Shanghai Aladdin Biochemical Technology Co., Ltd., Shanghai, China
Butein	487-52-5	$C_{15}H_{12}O_5$	272	Chalcones	Sigma-Aldrich(Shanghai) Trading Co., Ltd., Shanghai, China
Isoliquiritigenin	961-29-5	$C_{15}H_{12}O_4$	256	Chalcones	Sigma-Aldrich(Shanghai) Trading Co., Ltd., Shanghai, China
Formononetin	485-71-3	$C_{16}H_{12}O_4$	268	Isoflavones	Sigma-Aldrich(Shanghai) Trading Co., Ltd., Shanghai, China

Table S5. Source, identification and retail price information of 60 batch samples.

No.	Batch	Source of product	Identification information	Retail Price(CN￥)	No.	Batch	Source of product	Identification information	Retail Price(CN￥)
1	No. 1	Huazhou	*D. odorifera* heartwood	280	31	No. 2	Huazhou	*D. odorifera* heartwood	80
2	No. 1	Haikou	*D. odorifera* heartwood	260	32	No. 2	Haikou	*D. odorifera* heartwood	200
3	No. 1	Qingyuan	*D. odorifera* heartwood	300	33	No. 2	Qingyuan	*D. odorifera* heartwood	200
4	No. 1	Wanning	*D. odorifera* heartwood	260	34	No. 2	Wanning	*D. odorifera* heartwood	200
5	No. 1	Xinyi	*D. odorifera* heartwood	350	35	No. 2	Xinyi	*D. odorifera* heartwood	200
6	No. 1	Huazhou	*D. odorifera* heartwood	200	36	No. 2	Huazhou	*D. odorifera* heartwood	320
7	No. 1	Haikou	*D. odorifera* heartwood	200	37	No. 2	Haikou	*D. odorifera* heartwood	400
8	No. 1	Qingyuan	*D. odorifera* heartwood	300	38	No. 2	Qingyuan	*D. odorifera* heartwood	350
9	No. 1	Wanning	*D. odorifera* heartwood	260	39	No. 2	Wanning	*D. odorifera* heartwood	260
10	No. 1	Xinyi	*D. odorifera* heartwood	350	40	No. 2	Xinyi	*D. odorifera* heartwood	350
11	No. 1	Huazhou	*D. odorifera* heartwood	80	41	No. 3	Xinyi	*D. odorifera* heartwood	350
12	No. 1	Haikou	*D. odorifera* heartwood	60	42	No. 3	Huazhou	*D. odorifera* heartwood	200
13	No. 1	Qingyuan	*D. odorifera* heartwood	80	43	No. 3	Haikou	*D. odorifera* heartwood	260
14	No. 1	Wanning	*D. odorifera* heartwood	200	44	No. 3	Wanning	*D. odorifera* heartwood	260
15	No. 1	Xinyi	*D. odorifera* heartwood	200	45	No. 3	Qingyuan	*D. odorifera* heartwood	300
16	No. 1	Huazhou	*D. odorifera* heartwood	350	46	No. 3	Xinyi	*D. odorifera* heartwood	200
17	No. 1	Haikou	*D. odorifera* heartwood	200	47	No. 3	Huazhou	*D. odorifera* heartwood	200
18	No. 1	Qingyuan	*D. odorifera* heartwood	350	48	No. 3	Haikou	*D. odorifera* heartwood	200
19	No. 1	Wanning	*D. odorifera* heartwood	260	49	No. 3	Wanning	*D. odorifera* heartwood	260
20	No. 1	Xinyi	*D. odorifera* heartwood	300	50	No. 3	Qingyuan	*D. odorifera* heartwood	300
21	No. 2	Huazhou	*D. odorifera* heartwood	320	51	No. 3	Xinyi	*D. odorifera* heartwood	200
22	No. 2	Haikou	*D. odorifera* heartwood	260	52	No. 3	Huazhou	*D. odorifera* heartwood	100
23	No. 2	Qingyuan	*D. odorifera* heartwood	300	53	No. 3	Haikou	*D. odorifera* heartwood	60
24	No. 2	Wanning	*D. odorifera* heartwood	260	54	No. 3	Wanning	*D. odorifera* heartwood	200
25	No. 2	Xinyi	*D. odorifera* heartwood	300	55	No. 3	Qingyuan	*D. odorifera* heartwood	200
26	No. 2	Huazhou	*D. odorifera* heartwood	320	56	No. 3	Xinyi	*D. odorifera* heartwood	350
27	No. 2	Haikou	*D. odorifera* heartwood	180	57	No. 3	Huazhou	*D. odorifera* heartwood	350
28	No. 2	Qingyuan	*D. odorifera* heartwood	200	58	No. 3	Haikou	*D. odorifera* heartwood	60
29	No. 2	Wanning	*D. odorifera* heartwood	260	59	No. 3	Wanning	*D. odorifera* heartwood	260
30	No. 2	Xinyi	*D. odorifera* heartwood	200	60	No. 3	Qingyuan	*D. odorifera* heartwood	300
